# Controlled direct effect of psychiatric disorders on cardiovascular disease: evidence from a large Kurdish cohort

**DOI:** 10.1186/s12872-020-01794-6

**Published:** 2020-12-01

**Authors:** Zahra Darabi, Farid Najafi, Roya Safari-Faramani, Yahya Salimi

**Affiliations:** 1 Department of Epidemiology, School of Public Health, Kermanshah University of Medical Sciences, 6719851351 Kermanshah, Iran; 2 Research Center for Environmental Determinants of Health, Health Institute, Kermanshah University of Medical Sciences, 6719851351 Kermanshah, Iran; 3 Department of Epidemiology, Research Center for Environmental Determinants of Health, Health Institute, Kermanshah University of Medical Sciences, 6719851351 Kermanshah, Iran; 4 Social Development and Health Promotion Research Center, Health Institute, Kermanshah University of Medical Sciences, 6719851351 Kermanshah, Iran

**Keywords:** Cardiovascular disease, Psychiatric disorders, Oral and dental hygiene, Controlled direct effect

## Abstract

**Background:**

Psychiatric disorders are significantly associated with the incidence and prevalence of cardiovascular diseases, mortality, hospital readmissionn. Oral and dental hygiene may play a role in such association. This study aimed to evaluate the controlled direct effect of psychiatric disorders on cardiovascular diseases by controlling the mediating effect of oral and dental hygiene.

**Methods:**

The data used for this study came from the baseline phase of Ravansar Non-communicable Disease (RaNCD) cohort study. RaNCD cohort study is including a representative sample of 10,065 adults (35–65 years old) living in Ravansar, a city in the west of Iran. The marginal structural model with stabilized inverse probability weights accounted for potential confounders was used to estimate the controlled direct effect of psychiatric disorders on cardiovascular diseases. Three different models using three mediators including oral and dental hygiene behaviors, oral ulcer and lesions, and decayed, missing, and filled tooth, were used.

**Results:**

Psychiatric disorders increase the odds of cardiovascular diseases by 83% (OR = 1.83, CI 1.27, 2.61) and about two times (OR = 2.14, 95% CI 1.74, 2.63) when controlled for oral and dental hygiene behaviors, and oral ulcer and lesions as mediators, respectively. When decayed, missing, and filled tooth, as a mediator, was set at ≤ 8, there was no statistically significant controlled direct effect of psychiatric disorders on cardiovascular diseases (OR = 0.90, 95% CI 0.62, 1.30).

**Conclusion:**

Our results suggested that psychiatric disorder was directly related to cardiovascular diseases even if it was possible to have good oral and dental hygiene. The results suggested that interventions targeting people with psychiatric disorders could reduce prevalence of the cardiovascular diseases.

## Background

Cardiovascular disease (CVD) is the leading cause of death and morbidity worldwide and accounting globally for around 30% of all deaths [1]. Many effective interventions for primary and secondary prevention of heart disease rely on identifying people at risk. Despite the reliability of risk prediction models, a significant proportion of CVD cases occur in individuals without known risk factors [2]. Studies have shown that negative emotions, along with psychiatric disorders such as depression, can increase the CVD incidence and mortality [3–5]. Indeed, in people with depression, it is twice as likely to develop myocardial infarction as compared with the general population [6, 7]. Death from heart disease among depress people occurs even more than suicide deaths [6].

Several factors may have a role in the relationship between depression and CVD. Oral and dental hygiene is an indicator of general health and quality of life [8]. Such factors can affect quality of life through functional impairment, pain, discomfort and disability. Oral and dental problems are constantly highlighted in the list of common disease, but they are still referred to as silent epidemics and are therefore frequently ignored in public health planning [9, 10]. It has been shown that poor oral and dental hygiene including periodontal disease and teeth decay are associated with an increased risk of CVD [11, 12]. A review of 42 self-reported studies in 2012, revealed a correlation between periodontal disease and, atherosclerotic vascular disease [11]. Also, the harmful effects of mental disorders on organic control of human tissues, known as psychosomatic disorders, affect both periodontium and oral cavity in two ways: first, by creating unhealthy habits that are destructive for periodontal invasion, and secondly through the direct effect of the nervous system on the physiological balance of tissue [13]. Although there is large number of studies on the risk factors of CVD, but research on interaction between factors such as depression, oral and dental hygiene, and their mechanism are scarce [11, 14]. Therefore, we aimed to disentangle the direct effect of psychiatric disorder on CVD, controlling for oral and dental hygiene using a large sample of Ravansar Non-Communicable Disease (RaNCD) cohort study.

## Methods

The data consists of 10,065 individuals aged 35–65 years old from first cohort study in Kurdish region. In fact, RaNCD cohort study is one of the centers of the nationwide Prospective Epidemiological Research Studies in IrAN (PERSIAN). Inclusion criteria for participant recruitment were: age 35–65 years, minimum residence of 1 year in Ravansar city, likelihood of staying in Ravansar city in future, the willingness to participate and provision of written informed consent; having Iranian citizenship. After a census from total population of Ravansar district, 10,000 people aged 35–65 years old were sampled from both urban and rural areas. Data were collected using trained research assistants, who had good communication in Kurdish and local languages, conducted a door-to-door survey of all residents in urban areas to register their home address and to specify a code for each household. In the rural areas, local health units (Health Houses) already had all registration details for all residents. Household members’ details, including name, age and relationship, were registered, along with their contact number. All the process, questionnaires and forms for data collection have been verified by a central team responsible for designing and conduction the PERSIAN cohort study. Details of the sampling techniques, rational and methods are described elsewhere [15, 16].

### Measures

The information required for conducting this study was collected by standard questionnaires and by trained experts in the RaNCD cohort study. After the pre-pilot, measuring tools and protocols were revised to improve the validity and reliability of the study, as well as to streamline data collection. While the central team of PERSIAN cohort supervise all centers by a quality control team, the quality of all measurements and process are under supervision of an epidemiologist responsible for quality control in RaNCD. Although most of measurements have been collected by self-report, the medical history and the type of received treatments have been checked by interviewers and general physicians. Validity and reliability of some of measurements have been reported elsewhere [17–20]. The principal investigators decided to use the online version of the questionnaire, in accordance with the PERSIAN cohort protocol [16]. Procedures for data access, information on collaborations, questionnaire information, publications and other details can be found at http://persiancohort.com

### Exposure

Data on psychiatric disorders was collected based on self-report of any physician diagnosis. In addition, after consultation with psychiatrists, any medications related to psychiatric disorders were separated as psychiatric disease. Finally, the variable of psychiatric disorder was made up of a combination of four variables, including self-reported depression (diagnosed by a physician), taking antidepressant medication, self-reported other psychiatric disorders (diagnosed by a medical doctor), and taking medications for other psychiatric disorders.

### Outcome

Participants who had at least 1 of the following condition were classified as patients with CVD: a history of ischemic heart disease, heart failure and angina, stroke, and/or use of relevant medications for such condition. A list of used medications was also approved by consulting with an internal medicine specialist. In other words, people with CVD were those who either had a diagnosis of heart disease according to their statements by the doctor or had approved medicine for heart disease in their list of medicine.

### Mediators

Oral and dental hygiene was measured by asking the number of decayed, filled and missing teeth, oral lesions and ulcers, and hygiene behaviors; including frequency of daily teeth brushing, using dental floss and mouthwash. We used this data to create 3 oral and dental hygiene indices. Good oral hygiene behaviors were defined as cleaning teeth by brushing at least twice and flossing once daily [21]. We used self-reported data to evaluate the variable of ulcer and oral lesions. Decayed, missing, filled tooth (DMFT) was categorized to mild decay (DMFT ≤ 8) and severe decays (DMFT > 8) [22].

### Covariates

Demographic data including age and sex and socioeconomic status (SES) was collected by trained interviewers. The SES was measured by an asset index, constructed using principal component analysis from data on durable household assets [23, 24].

The blood pressure was measured using a manual sphygmomanometer (Riester). From each arm, blood pressure measured two times (with an interval of 10 min) and the average of two measurements was the final blood pressure of the participants. Data on smoking, alcohol and narcotics were collected, using questionnaires for personal habits (use of alcohol and tobacco). People who smoked more than 100 cigarettes in their lifetime or who were smoking daily, considered as smokers.

To measure and evaluate the physical activity, we used the International Physical Activity Questionnaire (IPAQ), which included questions about physical activity in the last 7 days [25]. The total Metabolic Equivalent of Task (METs) was calculated according to the questionnaire's instructions. For this study, physical activity was categorized to three-level: low physical activity (less than the 36.5 Met-hour/week), medium physical activity (between 36.6 and 44.9 Met-hour / week) and group with high physical activity (45 Met-hour/week and more) [26]. Levels of plasma total cholesterol and triglyceride were measured enzymatically.

The evaluation of food intake for each individual was done, using the Food Frequency Questionnaire (FFQ), which its validity and reliability previously was approved [25]. The questionnaire consisted of a list of 168 food items with standard portion size. The amounts indicated for each meal were converted to the recommended daily intake [27]. In this study, the variables of sugar and dairy intake were used in the form of g/day and vitamin D intake as μg/day. To evaluate the variables of sleep, the 24-h sleep time was calculated.

### Data cleaning

First, the data were modified in terms of the names of variables, codes and labels related to each variable and their unit of measurement. Then the qualitative data were identified and converted into appropriate codes based on the objectives of the plan. Then, for each variable, the outgoing data were identified and modified. First, the natural range of the data was determined. Then the data whose value was higher than expected became the highest natural number and the data whose value was lower than expected became the lowest natural number for the variable.

### Exposure-induced mediator-outcome confounders

**Fig. 1:**
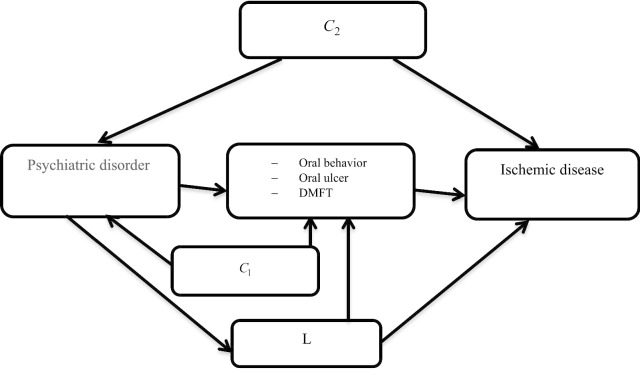
DAG of the hypothesized causal pathway between psychiatric disorder and CVD. DMFT: Decayed, Missing, Filled Tooth, DAG: Directed Acyclic Graph, *c*_1_ (confounder1): Age, Sex, Smoking, Educational, Diabetes mellitus, *c*_2_ (confounder2): Age, Sex, Smoking, BMI, Physical activity, Diet, Sleep time, L (Exposure-induced mediator-outcome confounders): Age, sex, Hypertension, Diabetes mellitus, Physical activity, Drinking, Hypercholesterolemia, SES, education, diet, BMI, Smoking, Drug use, Sugar use

We used the Directed Acyclic Graph (DAG) for variable selection (Fig. 1). let L denote oral and dental hygiene–CVD confounders affected by the psychiatric disorder (including age, sex, high blood pressure, diabetes mellitus, physical activity, smoking, drugs and alcohol, blood cholesterol, SES, body mass index (BMI), level of education and diet), C_1_ is exposure-mediator confounders and C_2_ is exposure-outcome confounders. In the presence of exposure-induced mediator-outcome confounders, natural direct and indirect effects are not identified [28].

In this study, we estimated the controlled direct effect (CDE) using the marginal structural models under the following assumptions. First, the C_2_ variables are sufficient to eliminate the confounding effect in the relationship between psychiatric disorder and CVD. Second, C_1_ and L variables are sufficient to control the confounding effect in the relationship between the oral and dental hygiene and CVD [29]. This model does not directly use regression methods, but instead uses a weighting method called "inverse probability weighting".

The base of marginal structural models is to analyze the regression model Y, as outcome, on E, as exposure, and M, as mediator, but instead of controlling the confounders C and L with regression, they are controlled by weighting approach [30, 31]. Therefore, two sets of weights are needed: one for the psychiatric disorder as exposure (${W}_{i}^{E}$) and one for the oral and dental hygiene as mediator (${W}_{i}^{M}$). In addition, two important assumptions for the estimation of CDE are: (1) the relationship between psychiatric disorder and CVD is dependent on C_2_ (2) the relationship between the oral and dental hygiene and CVD depends on C_1_ and L. Marginal Structural Models for a single mediator model allowing interaction between exposure and mediator is written as follows:

$$E\left[\left.Y\right|a,m\right]={\gamma }_{0}+{\gamma }_{1}e+{\gamma }_{2}m+{\gamma }_{3}em$$

In this study, each participant (i) is weighted by the total weight of exposure and mediator: ${{W}_{i}=W}_{i}^{E}\times {W}_{i}^{M}$. Then, the controlled direct effect can be obtained based on the estimates using such weighting approach. To calculate the CDE of E on Y when the mediator M = m, the following formula will be used if the exposure change from the level e* to the level e:

$$\begin{aligned} & CDE\left( m \right) = \gamma_{1} \left( {e - e^{*} } \right) + \gamma_{3} m\left( {e - e^{*} } \right) \\ & {\text{and}}\quad e^{*} = 0\quad {\text{and}}\quad e = 1 \\ \end{aligned}$$

so, the formula can be written as follows:

$$CDE\left( m \right) = \gamma_{1} + \gamma_{3} m$$

### Sensitivity analysis

Inappropriate classification may overestimate the association between psychiatric disorder and CVD. Sensitivity analysis was used to measure the strength of the findings. Therefore, we conducted two sensitivity analyzes: one for different definitions of exposure, and another for various mediator definitions that were found to be consistent with our findings.

We also initially performed all analyzes, using primary data that included missing data. The data were then analyzed after multiple imputation. The maximum missing data were 2.79%. The results of both analyzes were similar. We decided to present the results of primary data including missing.

### Data analysis

Descriptive statistics were used for describing the data. The marginal structural model was used to estimate controlled direct effect (CDE) in the presence of interaction between psychiatric disorder and, oral and dental hygiene [29]. The controlled direct effect is the effect of psychiatric disorder through pathways that do not pass through oral and dental hygiene status, holding oral and dental hygiene status at a constant level across all individuals. In this study, our outcome variable was a binary variable. The mediator variables were both binary and quantitative. We introduced the categorical factors as dummy variables. Records with missing data were excluded because the amount of missing data was small less than 1% and assumed to be missing at random. All analyses were performed using codes provided by VanderWeele et al. [32] in Stata11 (StataCorp, College Station, TX, USA).

## Results

From total of 10,065 participants of RaNCD cohort study, 5200 (52.5%) were female, 2496 (24.7%) were illiterate and 9076 (90.2%) were married (Table 1). Cardiovascular disease (angina, myocardial infarction, stroke, transient ischemic attack) was detected in 13.73% (1382 people) of the study population. The proportion of CVD among the females (17.70%) was higher compared to the males (10.37%). People who were over 45 years had a higher prevalence of CVD. Also, people with lower levels of education and lower socioeconomic conditions had a higher prevalence of CVD.

**Table 1:** Description of cardiovascular diseases in subgroups of the independent variables

Independent variables	With CVD	Without CVD	Total	Odds ratio (95% CI)
	n (%)	n (%)	n (%)	
*Gender*				
Female	920 (64.47)	4280 (49.42)	5200 (52.54)	1.80 (1.52, 2.13)
Male	507(35.53)	4380 (50.58)	4887 (47.46)	
*Age*				
< 45 years	223 (15.87)	3777 (43.61)	4000 (39.58)	1.05 (1.04, 1.06)
≥ 45 years	1182 (84.13)	4883 (56.39)	6065 (60.42)	
*Years of education*				
Illiterate	570 (39.94)	1926 (22.24)	2496 (24.74)	1.02 (0.93, 1.11)
1–5 years	525 (36.79)	3325 (38.39)	3850 (38.17)	
6–9 years	170 (11.91)	1511 (17.44)	1681 (16.67)	
10-12 years	101 (7.07)	1171 (13.52)	1272 (12.61)	
> 13 years	61 (4.27)	727 (8.39)	788 (7.81)	
*Marital status*				
Married	1267 (90.17)	7809 (90.17)	9076 (90.18)	1.17 (1.06, 1.30)
Divorced	129 (9.18)	438 (5.05)	567 (5.63)	
Bachelor	9 (0.64)	413 (4.76)	422 (4.19)	
*Socioeconomic status*				
1	326 (22.95)	1681 (19.52)	2007 (20.1)	1.06 (1.00, 1.13)
2	298 (20.98)	1707 (19.82)	2005 (19.99)	
3	286 (20.14)	1720 (19.97)	2006 (20.0)	
4	273 (19.22)	1733 (20.12)	2006 (20.0)	
5	237 (16.69)	1769 (20.54)	2006 (20.0)	
*Smoking status*				
No	1100 (79.07)	6927 (80.13)	8027 (79.99)	1.10 (0.86, 1.40)
Current	124 (8.91)	1055(12.20)	1179 (11.75)	
Former	167 (12.00)	662(7.65)	829 (8.26)	
*Drinking*				
Yes	95 (6.75)	540 (6.23)	635 (6.30)	1.24 (0.91, 1.68)
No	1311 (93.25)	8119 (93.77)	9430 (93.7)	
*Drug use*				
Yes	39 (2.77)	260 (3.00)	299 (2.97)	1.01 (0.63, 1.60)
No	1367 (97.23)	8399 (97.00)	9766 (97.03)	
*Physical activity*				
High	292 (20.76)	1825 (21.08)	2117 (21.03)	1.00 (0.99, 1.00)
Moderate	697(49.57)	4250 (49.09)	4947 (49.17)	
Low	417 (29.65)	2582 (29.82)	2999 (29.80)	
*Body mass index (BMI)*				
< 18/5	25 (1.79)	152 (1.76)	177 (1.78)	1.01 (1.00, 1.03)
18/5–24/9	362 (25.96)	2380 (27.71)	2742 (27.48)	
25–29/9	606 (43.47)	3739(43.53)	4345 (43.52)	
≥ 30	401 (28.76)	2317(26.97)	2718 (27.22)	
*Hypertension*				
Yes	878 (62.80)	697 (8.06)	1575 (15.69)	12.78 (11.06, 14.77)
No	520 (37.19)	7945 (91.93)	8465 (84.31)	
*Diabetes mellitus*				
Yes	273(19.51)	546(6.34)	819 (8.19)	2.19 (1.79, 2.67)
No	1126 (80.48)	8054 (93.65)	9180 (91.81)	
*Serum cholesterol*				
≥ 200	956(66.06)	5864 (67.71)	6820 (32.52)	1.00 (0.99, 1.00)
< 200	491(33.93)	2796 (32.28)	3287 (67.48)	
*Serum triglyceride*				
≥ 150	968 (69.24)	5873 (68.27)	6841 (68.41)	0.99 (0.99, 0.99)
< 150	430 (30.75)	2729 (31.72)	3159 (31.59)	
*Renal failure*				
Yes	17 (1.21)	84 (0.97)	101 (1)	0.90 (0.47, 1.73)
No	1387 (98.78)	8566 (99.02)	9953 (99)	

Among people with CVD, 90.17% were married, 9.18% divorced, and 0.64% were bachelor. Among people with CVD 8.91% were smokers and 12% were former smokers. About 6.75% of patients were alcoholics. Also, 2.77% of patient were drug user. Among people with CVD, 20.76% had high physical activity, 28.76% were obese, 62.80% had hypertension, 19.51% had diabetes mellitus, 66.06% suffer from high serum cholesterol, 69.24% with high serum triglyceride and 1.21% had renal failure. The prevalence of CVD was also higher among those with obesity (28.76%) (Table 1).

Our mediation analysis showed that the CDE of psychiatric disorder on CVD controlling the oral hygiene behaviors at the population level (all have a good hygiene behavior), increases the odds of CVD by 83% (OR = 1.83, 95% CI: 1.27, 2.61). In the sensitivity analyses, our findings did change. Using oral ulcer and lesions as a mediator, the CDE of psychiatric disorder on CVD was 2.14 (95% CI: 1.74, 2.63). However, controlling for DMFT as a mediator did not show a significant effect (OR = 0.90, 95% CI: 0.62, 1.30) (Table 2).

**Table 2:** Controlled direct effects of psychiatric disorders on CVD, using different mediators

Mediators	Odds ratio	95% CI	
		LL	UL
Oral behavior	1.83	1.27	2.61
Oral ulcer	2.14	1.74	2.63
Decayed, missing, filled tooth (DMFT)	0.90	0.62	1.30

## Discussion

In this study, the role of oral and dental hygiene as a mediator of the association between psychiatric disorders and cardiovascular disease, in the population of RaNCD cohort study was investigated. Psychiatric disorders had a direct effect on CVD, controlled for well-known mediators [33]. Although the precise nature of the links between depression and coronary heart disease (CHD) have not yet been established, these links are being highlighted more and more frequently, first of all from an epidemiological point of view and then with regard to the etiological and clinical aspects in patients with both condition [34]. In fact, the thoughtful statistical analysis and the study design are among the major strengths of the present study. The analysis was based on the most recent advances in causal inference methods, and sensitivity analyses were conducted to test for inappropriate classification and missing data. In this study, the prevalence of CVD was 13.73%, which is significantly different from the national average of 32.2% [34]. This contrast can be due to differences in the definition of disease in studies. Our study evaluated CVD based on self-reporting and used medication, while the definition of disease in the study by Forouzanfar et al. [34] was based on the checklist. Also, this difference could be partly due to lower risk factors for heart disease and healthy lifestyle among the population of RaNCD cohort study.

According to the World Health Organization (WHO), lifestyle is a combination of behavioral patterns and individual habits throughout life, including nutrition, physical activity, stress, smoking, and the quality of sleep, which are due to socialization. In Ravansar, due to its traditional texture and cultural issues, many of these behavioral patterns have not changed in the same way as in large industrial cities. In this study, the prevalence of psychiatric disorders was 7.78%. Based on the results of the second national study on mental disorders in Iran, the estimated prevalence of mood disorders and anxiety disorders were 4.35%, and 8.31%, respectively [35], which is in agreement with ours. Studies have also been conducted in the regional and provincial levels. In a study conducted in Kermanshah province by Sadeghi et al. [36], which was conducted by screening and psychiatric interviewing questionnaire, the periodic prevalence of depressive disorder was 1.6%, which is in contrast to the results of this study. Part of this difference is due to the time of the study. Our study was conducted many years after the study be Sadeghi et al. [36], and the prevalence of the depressive disorder has changed considerably over such period. There are other reasons contributing to such differences in the results which variation in age of participants plus use of different questionnaire are two important factors

In this study, a controlled direct effect between psychiatric disorders and CVD was determined. This effect was estimated to be 1.83 while controlling for oral and dental hygiene behaviors. That is if the variable of oral and dental hygiene remains constant and the psychiatric disorders reaches from the level a * = 0 to a = 1 level, then the risk of cardiovascular disease will be 1.83-fold. After the control of oral ulcer and lesions, the direct effect of psychiatric disorders was estimated to be 2.14, which was statistically significant. That means if the variable of oral ulcer and lesions stays constant and the variable of psychiatric disorders change from a level of a * = 0 to a = 1 level, then the risk of developing the cardiovascular disease will be 2.14 times higher. This is due to the presence of the problem and impairment of the internal tissue of the mouth, which has a direct and strong connection with the blood and vascular system and ultimately can directly affect the cardiovascular function. Also, oral lesions are directly related to the mental status of individuals and the presence of impaired psychologic function can lead to problems in the oral cavity. Some systemic conditions, such as diabetes and physical-psychiatric disorders, such as depression and psychological stress, affect the periodontium. Psychosocial stresses have an important role in periodontal disease if they affect the host's immune response.

In addition, the controlled direct effect of psychiatric disorders on CVD, controlling the DMFT variable, is estimated to be 0.90. This means that if the DMFT variable remains constant and the psychiatric disorders change from a * = 0 to the level a = 1, then the risk of cardiovascular disease is reduced to 10%, which is not statistically significant. Logistic regression analysis showed that the low frequency of brushing is significantly associated with a high prevalence of DM (OR = 2.03). This indicates that the low frequency of brushing leads to an increase in the decayed and missing teeth, which in turn leads to an increase in the incidence of CVD. As a result, the reason for the mild estimation obtained in this section is that people who have been suffering from oral and dental problems and consequently, CVD, due to their non-compliance with oral and dental hygiene, by their own decision or the physician's recommendation, they have changed their behavior and reduced their cardiovascular problems. The findings of this study indicate that psychiatric disorders have a direct impact on CVD in midlife. This study helps to better understand the determinants of cardiovascular disease. Previous studies have shown that poorer oral health, especially dental problems, is an independent predictor of morbidity and mortality from cardiovascular disease [12, 37]. Even after extensive adjustment for recognized confounders, edentate participants had a significantly elevated risk of all-cause and CVD mortality, but not cancer mortality. This highlights the importance of oral health as a mediator in the direct link between psychiatric disorders and cardiovascular disease. When we keep oral health constant, it means that despite considering this variable as a mediator, we can still prove the strong effect of oral health on cardiovascular disease. This strong association between mental illness and cardiovascular disease has been proven in previous studies [38]. However, the direct controlled effect of psychiatric disorders on cardiovascular disease through oral health has not been previously reported in any article. Our findings confirm the previous evidence of the effects of mental health problems on chronic illnesses and suggest that reducing mental health problems may have lifelong benefits for the health of other organs of the body.

### Strengths and limitations

For the purpose of this study, we used baseline data from large cohort of Kurdish people. We also used causal inference methods for mediator analysis that could resolve the limitations of traditional mediation methods. Although the bias due to unmeasured confounding cannot be ruled out, we used DAG to find the all known variables that may be a confounder in the relationships. We assume that our marginal structural models were correctly specified and that the assumption holds. There are several limitations of our study. First, examinations for calculating oral and dental health have some drawbacks: the number of teeth in danger is not seen in this index, the reason for the loss of teeth is not easily detectable; the filled part is affected by the decision of the clinician to fill the tooth. Nowadays, in most cases, restorations are carried out with tooth-colored materials that are difficult to distinguish from the natural tissue of the tooth in epidemiological studies. Second, because of the cross-sectional nature of the data, it was not possible to establish temporality between exposure, mediator and outcome. The current evidence suggest that the association of CVD and psychiatric disorder is bidirectional, psychiatric disorder can cause CVD and CVD can cause psychiatric disorder [7, 39]. Third, we assume no unmeasured confounding of the exposure-outcome and mediator-outcome association. However, both CVD and psychiatric disorder may have a common cause such as inflammatory biomarkers that not studied in the current study. Fourth, the self-reported nature of psychiatric disorders and cardiovascular disease might lead to recall bias. Although, we have verified diagnosis for self-reporting data, using the list of taken medications.

## Conclusion

We demonstrate that psychiatric disorders are directly related to cardiovascular disease. In the optimal situation of hygiene behaviors and oral lesions in the presence of psychiatric disorders, there was a higher probability of having CVD, compared with the absence of psychiatric disorders. These results suggested that interventions targeting people with psychiatric disorders could reduce prevalence of the CVD.

## Data Availability

The datasets used during the current study are available from the corresponding author on reasonable request.

## Abbreviations

CVD: Cardiovascular disease
RaNCD: Ravansar Non-communicable Disease
PERSIAN: Prospective Epidemiological Research Studies in IrAN
DMFT: Decayed, missing, filled tooth
SES: Socioeconomic status
IPAQ: International physical activity questionnaire
MET: Metabolic equivalent of task
FFQ: Food frequency questionnaire
DAG: Directed acyclic graph
BMI: Body Mass Index
CDE: Controlled direct effect
CHD: Coronary heart disease
WHO: World Health Organization
DM: Diabetes mellitus
OR: Odds ratio
